# Impact of Nutrient Enrichment on Community Structure and Co-Occurrence Networks of Coral Symbiotic Microbiota in *Duncanopsammia peltata*: Zooxanthellae, Bacteria, and Archaea

**DOI:** 10.3390/microorganisms12081540

**Published:** 2024-07-27

**Authors:** Chuanzhu Bai, Qifang Wang, Jinyan Xu, Han Zhang, Yuxin Huang, Ling Cai, Xinqing Zheng, Ming Yang

**Affiliations:** 1 School of Environmental and Chemical Engineering, Shanghai University, Shanghai 200444, China; 15779226849@shu.edu.cn (C.B.); huangyuxin0120@163.com (Y.H.); 2 Key Laboratory of Marine Ecology Conservation and Restoration, Third Institute of Oceanography, Ministry of Natural Resources, Xiamen 361005, China; wangqifang@tio.org.cn (Q.W.); 202120949@mail.sdu.edu.cn (H.Z.); zhengxinqing@tio.org.cn (X.Z.); 3 Fujian Key Laboratory of Island Monitoring and Ecological Development (Island Research Center, MNR), Pingtan 350400, China; xujy_liesmars@whu.edu.cn; 4 Observation and Research Station of Island and Coastal Ecosystems in the Western Taiwan Strait, Ministry of Natural Resources, Xiamen 361005, China; 5 Fujian Provincial Station for Field Observation and Research of Island and Coastal Zone, Zhangzhou 363216, China

**Keywords:** reef-building coral, nutrient enrichment, coral symbiotic microorganism, microbial community, co-occurrence pattern

## Abstract

Symbiotic microorganisms in reef-building corals, including algae, bacteria, archaea, fungi, and viruses, play critical roles in the adaptation of coral hosts to adverse environmental conditions. However, their adaptation and functional relationships in nutrient-rich environments have yet to be fully explored. This study investigated *Duncanopsammia peltata* and the surrounding seawater and sediments from protected and non-protected areas in the summer and winter in Dongshan Bay. High-throughput sequencing was used to characterize community changes, co-occurrence patterns, and factors influencing symbiotic coral microorganisms (zooxanthellae, bacteria, and archaea) in different environments. The results showed that nutrient enrichment in the protected and non-protected areas was the greatest in December, followed by the non-protected area in August. In contrast, the August protected area had the lowest nutrient enrichment. Significant differences were found in the composition of the bacterial and archaeal communities in seawater and sediments from different regions. Among the coral symbiotic microorganisms, the main dominant species of zooxanthellae is the C1 subspecies (42.22–56.35%). The dominant phyla of bacteria were Proteobacteria, Cyanobacteria, Firmicutes, and Bacteroidota. Only in the August protected area did a large number (41.98%) of SAR324_cladeMarine_group_B exist. The August protected and non-protected areas and December protected and non-protected areas contained beneficial bacteria as biomarkers. They were *Nisaea*, *Spiroplasma*, *Endozoicomonas*, and Bacillus. No pathogenic bacteria appeared in the protected area in August. The dominant phylum in Archaea was Crenarchaeota. These symbiotic coral microorganisms’ relative abundances and compositions vary with environmental changes. The enrichment of dissolved inorganic nitrogen in environmental media is a key factor affecting the composition of coral microbial communities. Co-occurrence analysis showed that nutrient enrichment under anthropogenic disturbances enhanced the interactions between coral symbiotic microorganisms. These findings improve our understanding of the adaptations of coral holobionts to various nutritional environments.

## 1. Introduction

Healthy coral reefs are among Earth’s most productive and biodiverse ecosystems [1], providing various ecosystem services to humans and performing essential functions [2]. However, the environment in coastal areas has been damaged globally by increased human activity, resulting in the degradation of many coral reefs [3,4]. Although many anthropogenic environmental stressors severely affect coral reef communities, the effects of nutrients on coral reefs are diverse [5]. Numerous studies have shown that the extent to which corals respond to nutrients depends on the type of nutrient enrichment. Nitrogen enrichment may reduce coral calcification levels but can increase the growth and development of Symbiodiniaceae to a certain extent. In contrast, phosphorus enrichment is more likely to increase the calcification rate of corals [6]. Appropriate ammonia acclimation can improve coral immunity [7]. Nitrate works synergistically with temperature [8], and nutrient enrichment reduces the ability of corals to resist bleaching [9]. Eutrophication also affects the distribution of allele frequencies with coral bleaching resistance [10]. Therefore, studying the adaptability of corals to complex nutrient-enriched environments could provide a theoretical basis and guidance for domesticating corals to withstand environmental stress.

Coral stress resistance and adaptability are closely related to coral microbiota, which includes symbiotic algae, bacteria, archaea, fungi, and viruses. This complex ecological assemblage is known as the coral holobiont [11]. Research suggests that microbial communities that have coevolved with their coral hosts may help corals adapt to their environments [12]. As the dominant microorganisms in coral symbionts, symbiotic zooxanthellae provide the most nutritional requirements for their coral hosts [13]. Research has shown that coral holobionts adapt to environmental changes primarily by reconfiguring zooxanthellae components [14]. Increasing ambient temperatures cause the coralline zooxanthella genus to shift from *Cladocopium* (formerly Clade C) to *Durusdinium* (formerly Clade D) [15]. Moderate nutrient levels can increase symbiotic algal density and improve coral photosynthesis [16]. Increasing evidence has revealed the critical role played by specific bacterial species in prokaryotic communities in maintaining the fitness of the entire holobiont [17]. This type of bacteria is called the “core bacteria” of corals and remains stable within a specific time and space range [18]. Different bacterial community compositions help corals cope with changes in sea-surface temperature and eutrophication [19,20]. Crenarchaeota are the most abundant archaea found in corals. These archaea are primarily involved in the recycling and transforming of nutrients in corals [21]. This transformation results in changes in the archaeal communities of corals as nutrients enter the environment. Nutrient changes significantly alter species richness and community variability among coral microbiomes [22]. The structure of coral microbial communities is critical to host health; as the commensal microbial community changes, the health status of corals may also change [23]. Environmental stress may allow other organisms to replace stable microbiota, leading to the development of coral diseases [24]. Furthermore, increased pathogenic microorganisms can destabilize coral self-regulatory systems [25]. Therefore, characterizing the structure of symbiotic coral microorganisms is critical for understanding coral reef resilience and predicting ecological change.

The coral communities in Dongshan Bay form the northernmost edge of the reef-building coral communities in China. Located near the shore, they are easily affected by anthropogenic activities under the combined stress of global climate change and regional nutrient enrichment. The overall coral community in Dongshan Bay showed low species richness (less than 10 species), with coral species usually having high environmental tolerance primarily dominated by slow-growing block corals or plate corals with large polyps [26]. This situation provides a unique context for exploring how stress-tolerant coral species respond to long-term nutrient enrichment, primarily through anthropogenic activities [27]. *Duncanopsammia peltata* (Esper, 1790) is widely distributed in the Indo-Pacific region and is a dominant coral species with ecological significance in Dongshan Bay [26]. Therefore, it can serve as a model species for understanding how corals adapt to changing environmental conditions [28]. A detailed analysis of the relationship between the coral symbiotic microorganisms and the environment in Dongshan Bay would be more conducive to the protection of corals. Previous studies have mainly elaborated on the effect of temperature on symbiotic coral microbial communities [29]. However, only some studies have focused on the response of symbiotic coral microorganisms to changes in nutrients. The present study aimed to comprehensively investigate the adaptation of *D. peltata* symbiotic microorganisms to nutrient enrichment by collecting *D. peltata* from different areas (protected and non-protected areas) at other times (August and December) in Dongshan Bay waters and analyzing the diversity, species composition, and co-occurrence of coral symbiotic microorganisms. High-throughput sequencing of a specific barcode region (ITS or 16S) was applied to determine the community composition and changes in zooxanthellae, bacteria, and archaea in *D. peltata* under different nutritional environments. Although high-throughput metabarcode sequencing has some potential limitations and challenges, such as limited resolution, PCR bias, and quantitative accuracy, it has significant advantages, such as high-throughput capability, wide applicability, high sensitivity, relative cost-effectiveness, and data reproducibility, which make it a powerful tool for the analysis of coral symbiotic microbial communities. We reveal the relationship between these different symbiotic microbial communities in response to environmental variations and identify the respective keystone taxa groups involved. This study shows the characteristics and functional relationships of community changes in coral symbiotic microorganisms under nutrient enrichment and provides a theoretical reference for coral ecological protection.

## 2. Materials and Methods

### 2.1. Sampling Area Description and Sample Collection

**Figure 1:**
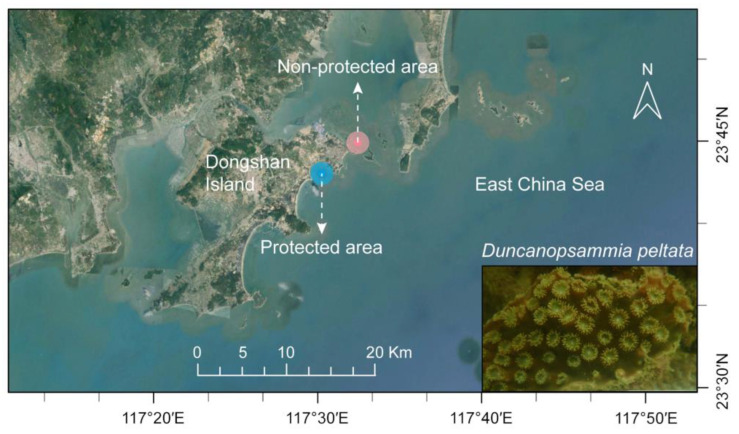
A map showing the sampling areas of Dongshan Bay, including protected and non-protected areas, where *Duncanopsammia peltata* predominates.

Scuba divers collected *D. peltata* in the summer (August 2022) and winter (December 2022) from sampling areas with different degrees of eutrophication in Dongshan Bay, China (Figure 1), including a protected area (PA, sampling at 23.71° N, 117.50° E), approved as a coral nature reserve since 1997, and a non-protected area (NPA, sampling at 23.73–23.76° N, 117.49–117.55° E). Three individual coral samples from fringing reefs at each sampling site, as well as their surrounding water samples (3 replicates), and surface sediment (0–2 cm below the surface) samples (3 replicates), were collected at the same time. Coral, water, and surface sediment samples (3 replicates for each) were collected from each PA and NPA in December (DecPA and DecNPA). The coral samples were cut into 1 cm^3^ cubes, placed in a 2 mL enzyme-free cryovial, and quickly frozen in liquid nitrogen. Five liters of seawater were filtered through three 0.22 μm mixed fiber membranes (50 mm, Aquo-system, Millipore) to collect microorganisms. Filtered seawater samples (approximately 500 mL) were stored in polyethylene (PE) bottles and used to determine the dissolved inorganic nutrients, including phosphate, nitrate, nitrite, ammonium, and dissolved inorganic nitrogen (DIN). The sediments were placed in 3 × 5 mL and 3 × 50 mL enzyme-free tubes. All samples were shipped back to the laboratory on dry ice and stored at −80 °C until experimental processing.

### 2.2. Environmental Parameters

Seawater was collected in PE bottles, and the nutrient salt concentration was determined using the national standard colorimetric method within 12 h. The measurement accuracy is within ±10%. Dissolved oxygen (DO), salinity, turbidity, temperature, pH, and chlorophyll a (Chl a) were obtained through station buoy monitoring (taking data within a month of sampling), and a pre-calibrated YSI EXO2 multi-parameter radiosonde (Xylem, Washington, DC, USA) was used to measure these parameters in situ. Total phosphorus (TP), total nitrogen (TN), and total organic carbon (TOC) in the sediments were measured by Yanqu Information Technology Co., Ltd., Hangzhou, China. The TOC, TP, and TN testing method standards were LY/T 1237-1999 [30], HJ 717-2014 [31], and NY/T 88-1988 [32], respectively.

### 2.3. DNA Extraction and Sequencing

Coral tissue (1 g) was ground using a liquid nitrogen precooled mortar and total coral microbial genomic DNA was extracted using the E.Z.N.A.^®^ soil DNA Kit (Omega Bio-tek, Norcross, GA, USA) according to the manufacturer’s instructions. We used the Power Soil DNA Isolation Kit (MoBio, Carlsbad, CA, USA) for the seawater filter membrane samples and the Fast DNA SPIN Kit (MP Biomedical, Santa Ana, CA, USA) to extract total DNA from sediment samples (1 g). The quality and concentration of DNA were determined using 1.0% agarose gel electrophoresis using a NanoDrop2000 spectrophotometer (Thermo Scientific, Waltham, MA, USA) and kept at −80 °C for storage for further use. DNA samples were used to sequence symbiotic algal internal transcribed spacer ITS rRNA genes and bacterial and archaeal 16S rRNA genes. The corresponding primers (Table S1) were used to amplify the hypervariable regions of zooxanthellae, bacteria, and archaea. The amplification conditions for algal amplicon PCR started with denaturation at 95 °C for 3 min, followed by 27 cycles at 95 °C for 30 s, 55 °C for 30 s, and 72 °C for 45 s, and single extension at 72 °C for 10 min, ending at 4 °C. The PCR amplification procedure for bacteria and archaea included 30 cycles of initial denaturation at 95 °C for 5 min, denaturation at 95 °C for 1 min, annealing at 60 °C for 50 s, amplification at 72 °C for 50 s, and a final extension at 72 °C for 7 min.

The PCR amplification system contained 5 μL 5 × Q5@ Reaction Buffer, 5 μL 5 × Q5@ High GC Enhancer, 0.75 μL (2.5 mM) dNTPs, 0.75 μL each of upstream and downstream primers (10 µM), 0.1 μLQ5@ High-Fidelity DNA Polymerase, 25 ng of DNA template, and ddH_2_O added to a volume of 25 μL. The PCR product was extracted from 2% agarose gel, purified using the PCR Clean-Up Kit (YuHua, Shanghai, China) according to the manufacturer’s instructions, and quantified using Qubit 4.0 (Thermo Fisher Scientific, USA). The sequencing platform was an Illumina MiSeqPE300 (measured by Genedenovo Biotechnology Co., Ltd., Guangzhou, China, and Majorbio Bio-Pharm Technology Co., Ltd., Shanghai, China).

### 2.4. Sequence Analysis

The DADA2 plugin in the Qiime2 pipeline was used to denoise the high-quality sequences. The denoised sequences are known as amplicon sequence variants (ASVs). To minimize the impact of sequencing depth on the diversity data analysis, all sample sequences were flattened according to the minimum number of sample sequences—the average sequence coverage after sample sequence smoothing was guaranteed to reach 99%. Species taxonomic analysis of ASVs was performed using the naive Bayes consensus taxonomic classifier in Qiime2 based on the Sliva 16S rRNA gene database. The Symbiodiniaceae taxonomy was carried out at http://sym-its2.marinegenomics.cn/Delineation (accessed on March 2023) [33].

### 2.5. Data Analysis

Bioinformatics analysis was performed using the Majorbio Cloud platform (https://cloud.majorbio.com). Mothur (Version 1.35.1) software calculates the microorganisms’ alpha diversity (Chao 1, Shannon index). Welch’s *t*-test was used to determine the difference in alpha diversity between the groups.

Non-metric multidimensional scaling analysis (NMDS) based on the Bray–Curtis distance algorithm was used to determine the similarity of microbial communities between the sample groups. The Wilcoxon rank-sum test of the STAMP software (v2.1.3) was used to determine whether there were significant differences in the species composition of the microbial community. Linear discriminant analysis (LDA; score > 2, *p* < 0.05) was used to identify microbial biomarker taxa that caused significant differences between samples. Mantel tests were performed using the “vegan (2.6-6.1)”, “stats (3.6.2)”, “ggplot2 (3.5.1)”, “ggrepel (0.9.5)”, “extrafont (0.91)”, and “ggpubr (0.6.0)” packages in R (version 4.3.0) to reveal Pearson correlations between coral microbial communities and environmental parameters. In the co-occurrence network analysis, seawater and sediments were divided into AugPA, AugNPA, DecPA, and DecNPA, whereas coral symbiotic microorganisms were divided into PA, NPA, August (Aug.), December (Dec.), and whole (whole) sample divisions. Co-occurrence networks were conducted using the “igraph (2.0.3)” and “hmisc (5.1-3)” packages of R and visualized using Gephi software (version 0.10.1). The network was constructed based on genus relative abundance >0.01%, Spearman’s |ρ| > 0.6, *p* < 0.01 [34]. The relationships between symbiotic coral microorganisms can be determined by comparing the topological structures of the networks.

## 3. Results

### 3.1. Physico-Chemical Properties in the Surrounding Sediment and Water

The sediment samples from PA had significantly lower TOC, TP, and TN contents than NPA sediment samples in August and December (Table 1). According to the continuous monitoring data from adjacent buoys, the turbidity in the PA was significantly lower than that in the NPA in August and December, with higher DO concentrations. Consistently, the water samples collected from the PA in August had lower DIN and DIP levels than those from the NPA. However, in December, high levels of nutrients were observed unexpectedly in water samples from both the NPA and PA, possibly due to the temporary environmental variation in the PA. Based on a multi-year monitoring study on the degree of eutrophication in Dongshan Bay assessed using the modified potential eutrophication assessment model-PE3 [35], the average eutrophication degree of the bay where the NPA sampling site was located was 5 in 2008 (light eutrophication), 6 in 2019 (medium eutrophication), and 4 in 2020 (potential eutrophication), with relatively high DIN and DIP, consistent with our observed data. According to the assessment model (Table S2), the degree of eutrophication of our sampling sites was 2 for both AugPA and AugNPA (medium nutrition and N-limiting) and 5 for DecPA and DecNPA (light eutrophication). Nalley et al. (2023) [16] found that DIN exceeding 10 μM inhibits the growth, development, and reproduction of corals, which can serve as an indicator of nutrient enrichment stress for coral, suggesting that except for the sample from the PA in August, the other samples were under nutrient enrichment stress.

### 3.2. Characteristics of Microbial Communities in Surrounding Sediment and Water

The difference in the alpha diversity of PA and NPA sediments (Figure S1) was not statistically significant (*p* > 0.05). In contrast, the archaeal alpha diversity of AugNPA was significantly higher than that of AugPA (*p* < 0.05). In seawater, the bacterial alpha diversity of DecPA was considerably higher than that of DecNPA (*p* < 0.05). In contrast, there was no significant difference in the archaeal alpha diversity between DecPA and DecNPA (*p* > 0.05).

The species compositions of sediment bacteria and archaea of AugPA were significantly different from those of the other three groups (Figure S2), with the most significant difference observed for archaea. The bacterial and archaeal species compositions of the seawater (Figures S3 and S4), DecPA, and DecNPA were significantly different; however, the bacterial differences were more significant. These findings support the alpha diversity results.

To reveal the intergroup differences among samples from different regions, beta diversity analysis was performed using NMDS and PCoA (Figures S5 and S6). The NMDS (stress = 0) results showed that the bacterial and archaeal community structures of the samples from different areas were separated regardless of whether they were sediment or seawater. This indicates that the environmental differences in the various regions were significant, and there were noticeable intergroup differences among the samples.

### 3.3. Diversity and Composition of Zooxanthellae Communities in Coral Symbiotic Microorganisms

In the coral zooxanthellae ITS gene sequencing of the 12 samples, 162,855 high-quality sequences were obtained, with an average of 13,571 sequences per sample (SD = 4260). After sequence denoising, 88 ASVs were identified (Supplementary Table S3).

**Figure 2:**
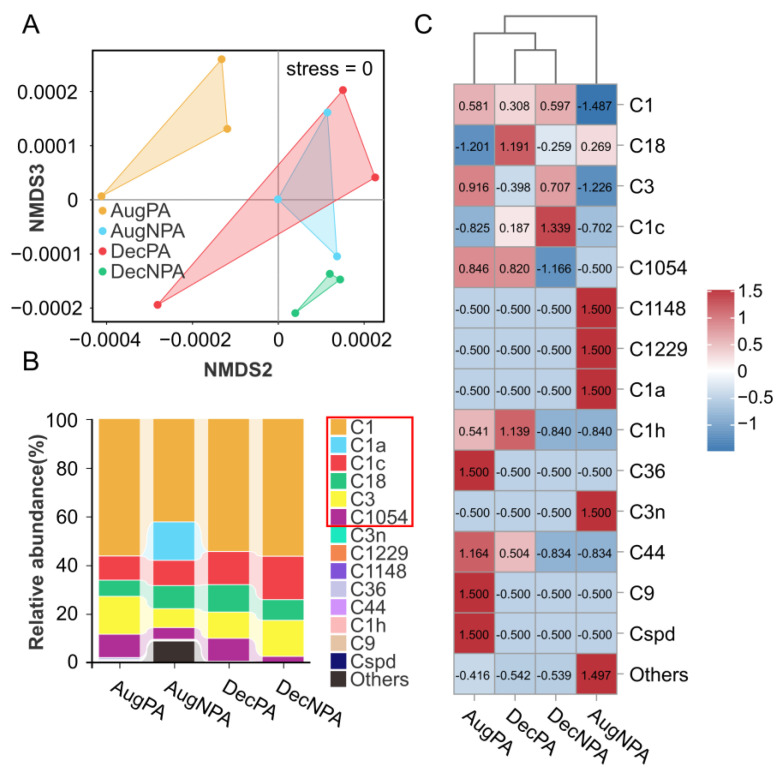
Beta diversity analysis (**A**), species composition (**B**), and species difference heat map (**C**) of coral symbiotic zooxanthellae in the protected area and non-protected area in August (AugPA and AugNPA) and December (DecPA and DecNPA).

The NMDS (stress = 0) analysis of coral zooxanthellae showed significant intergroup differences between AugPA, AugNPA, DecPA, and DecNPA (Figure 2A). Fourteen symbiotic species from *Cladocopium* were found in all samples (Figure 2B). Together, these dominant algae (relative abundance exceeding 1%) accounted for 90.50% to 99.96% of species, including C1 (42.22–56.36%), C1a (15.81%), C1c (9.89–17.81%), C18 (accounting for 6.66–11.27%), C3 (7.78–15.57%), and C1054 (2.50–9.60%). Among these, the dominant alga C1a is endemic to AugNPA. Among non-dominant species (Figure 2C), C36, C9, and Cspd were more abundant in AugPA than in the other samples. The abundances of C1h and C44 in AugPA and DecPA were lower than those in AugNPA and DecNPA, respectively. In addition, compared with AugPA, DecPA, and DecNPA, the abundances of C1148, C1229, and C3n were the highest in AugNPA.

### 3.4. Diversity and Composition of Bacterial Communities in Coral Symbiotic Microorganisms

In the 16S rRNA gene sequencing of coral bacteria, six samples collected in August produced 226,206 high-quality sequences with an average of approximately 37,701 sequences per sample (SD = 7222). After sequence denoising, 3314 ASVs and 40 bacterial phyla were identified. Similarly, among the six samples collected in December, 219,633 high-quality sequences were obtained with an average of approximately 36,605 sequences per sample (SD = 19,804). After denoising, 8889 ASVs and 53 bacterial phyla were identified (Table S4).

**Figure 3:**
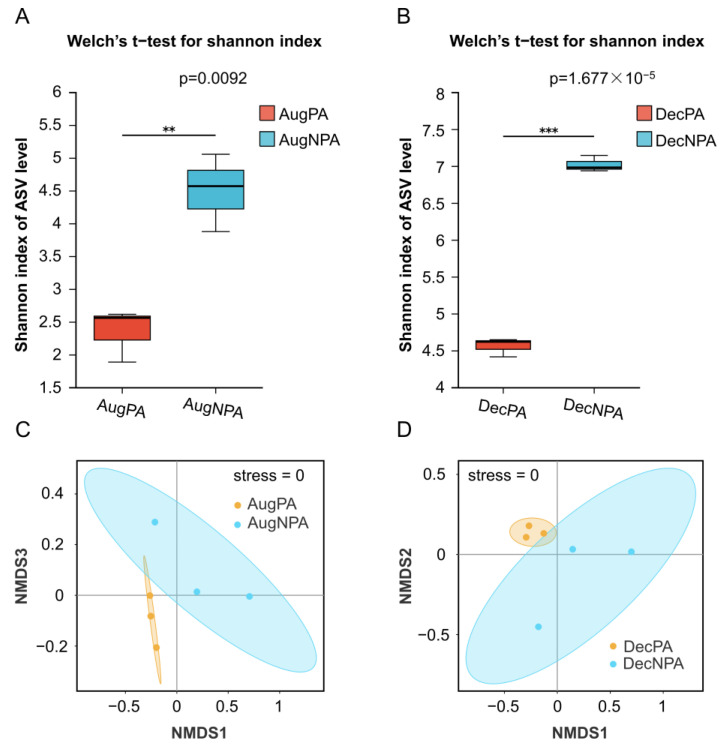
Shannon index (**A**,**B**) (***: *p* < 0.001, **: *p* < 0.01) and β diversity analysis (**C**,**D**) of coral symbiotic bacteria in the protected area and non-protected area in August (AugPA and AugNPA) and December (DecPA and DecNPA).

The Shannon index results for coral bacteria showed that the species diversity of the NPA was significantly higher than that of the PA (Figure 3A,B), with statistical significance (*p* < 0.01). Through NMDS analysis of the bacteria (stress = 0), significant intergroup differences were observed for AugPA, AugNPA, DecPA, and DecNPA (Figure 3C,D).

**Figure 4:**
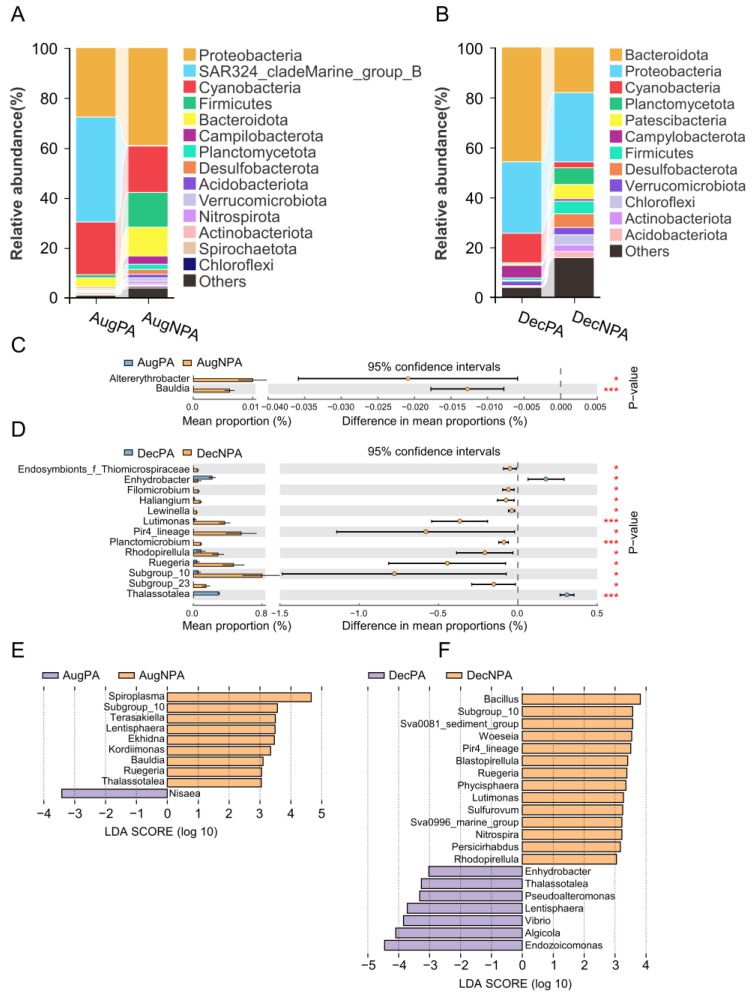
Coral symbiotic bacterial community composition at the phylum level (**A**,**B**), and the difference analysis (**C**,**D**) (***: *p* < 0.001, *: *p* < 0.05) and linear discriminant analysis (**E**,**F**) at the genus level in the protected area and non-protected area in August (AugPA and AugNPA) and December (DecPA and DecNPA).

The composition of the dominant bacterial groups (phylum level), that is, the bacterial groups with a relative abundance exceeding 1%, included Proteobacteria, SAR324_cladeMarine_group_B, Cyanobacteria, Firmicutes, Bacteroidetes, Campylobacterota, Planctomycetota, Desulfobacterota, etc. (Figure 4A,B). Proteobacteria, Cyanobacteria, Firmicutes, and Bacteroidetes were the most abundant bacterial groups in all samples.

In the bacterial differential analysis (genus level), the abundance of *Altererythrobacter* and *Bauldia* was higher (*p* < 0.05 and *p* < 0.001) in AugNPA than in AugPA (Figure 4C). The abundances of *Lutimonas* and *Planctomicrobium* in DecNPA were higher than in DecPA (*p* < 0.001). In contrast, the abundances of *Enhydrobacter* and *Thalassotalea* in DecPA (*p* < 0.05 and *p* < 0.001, respectively) were higher than those in DecNPA (Figure 4D).

At the bacterial LDA (genus level), *Nisaea* in AugPA and *Spiroplasma* in AugNPA were identified as biomarkers (Figure 4E). *Endozoicomonas* of DecPA and *Bacillus* of DecNPA were identified as biomarkers (Figure 4F).

### 3.5. Diversity and Composition of Archaeal Communities in Coral Symbiotic Microorganisms

**Figure 5:**
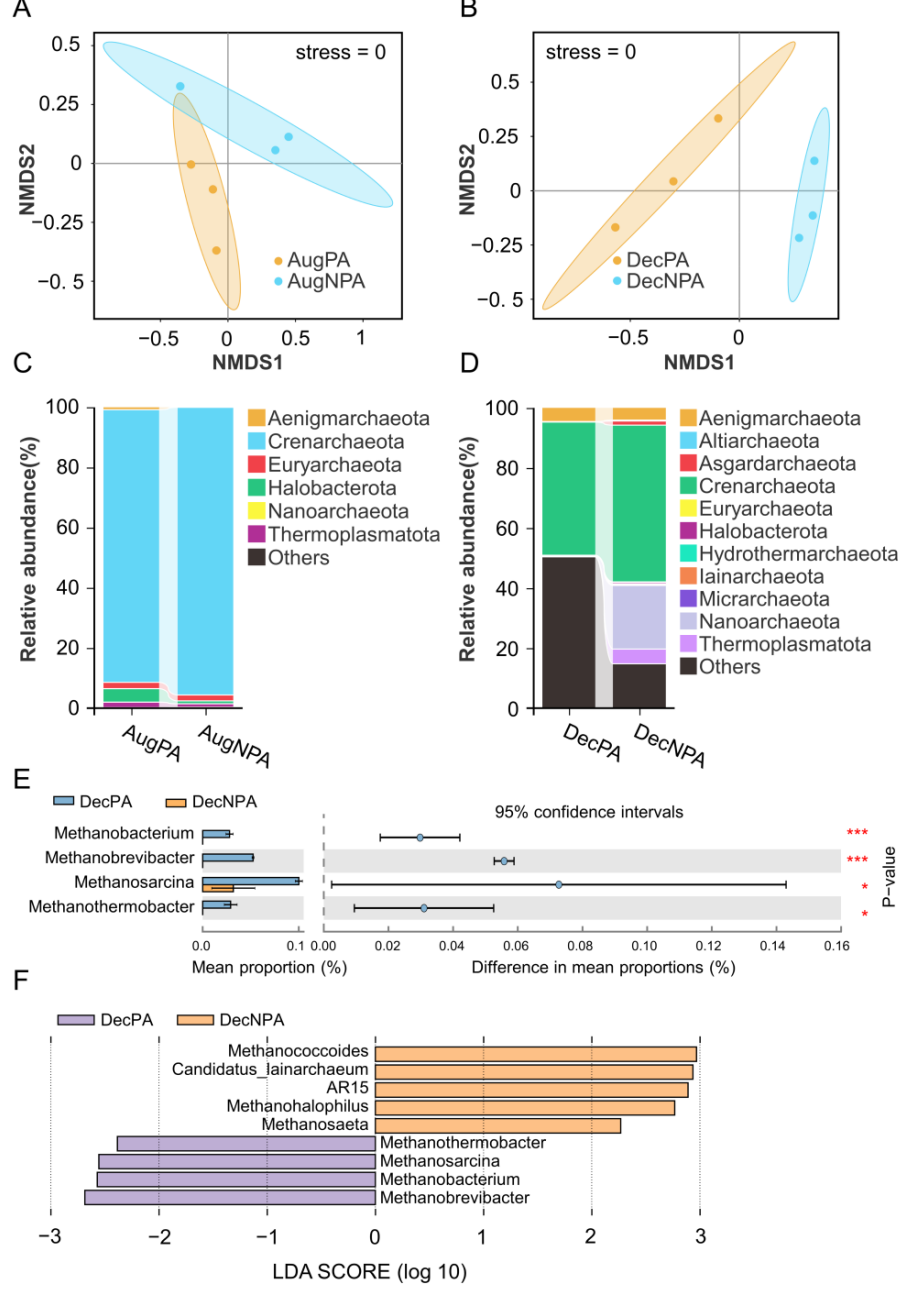
Coral symbiotic archaeal beta diversity analysis (**A**,**B**), community composition (**C**,**D**), and the difference analysis (**E**) (***: *p* < 0.001, *: *p* < 0.05) and linear discriminant analysis (**F**) in the protected area and non-protected area in August (AugPA and AugNPA) and December (DecPA and DecNPA).

In the 16S rRNA gene sequencing of coralline archaea, 58,200 high-quality sequences were obtained from six samples collected in August, with an average number of sequences per sample of 9700 (SD = 1998). After sequence denoising, 192 ASVs and 7 archaeal phyla were identified (Table S5). In December, 340,092 high-quality sequences were obtained from six samples, with an average number of sequences per sample of 56,682 (SD = 11,881). After sequence denoising, 1864 ASVs and 12 archaeal phyla were identified (Table S5). The difference in the archaeal Shannon indices between August and December was not statistically significant (Figure S7). According to the NMDS analysis of archaea (stress = 0), there were substantial differences between AugPA and AugNPA, as well as between DecPA and DecNPA (Figure 5A,B).

In terms of the species composition (phylum level) of the archaea, the dominant archaeal taxa (relative abundance > 1%) were Crenarchaeota, Euryarchaeota, Halobacterota, and Thermoplasmatota. The archaeal taxa with the highest relative abundance in all the samples were mainly Crenarchaeota (Figure 5C,D).

The species difference analysis of the August archaea showed no significant differences. In the species difference analysis at the genus level, the abundances of *Methanobacter* and *Methanobrevibacter* were higher (*p* < 0.001) in DecPA than in DecNPA (Figure 5E). The LDA of the August archaea revealed no biomarkers.

In the December archaea in the LDA (Figure 5F), the biomarker genera for DecPA were *Methanobrevibacter* and *Methanobacter*, whereas those for DecNPA were *Methanococcoides* and *Candidatus_lainarchaeum*.

### 3.6. Correlation Analysis between Microorganisms and Environmental Factors

**Figure 6:**
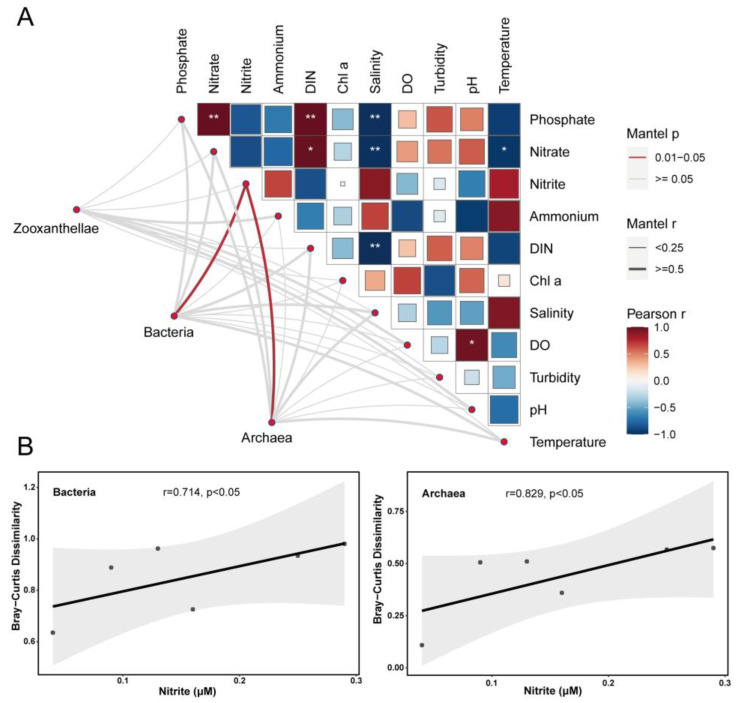
Mantel analysis between coral symbiotic microbial community beta diversity and environmental factors (**A**) (**: *p* < 0.01, *: *p* < 0.05); linear regression analysis of bacteria and archaea with nitrite (**B**).

By studying the correlation between coral symbiotic microorganisms (zooxanthellae, bacteria, and archaea) and environmental factors at the species or genus level, we found that coral symbiotic microorganisms were significantly related to nitrite in the DIN (Figure 6A). Linear regression analysis revealed that bacteria and archaea in the coral symbiotic microorganisms were significantly and positively correlated with nitrite levels (Figure 6B). The results indicate that bacteria and archaea in coral symbiotic microorganisms may play a critical role in the adaptation of coral holobiont to nitrogen nutrient enrichment.

### 3.7. Co-Occurrence Network Analysis

**Figure 7:**
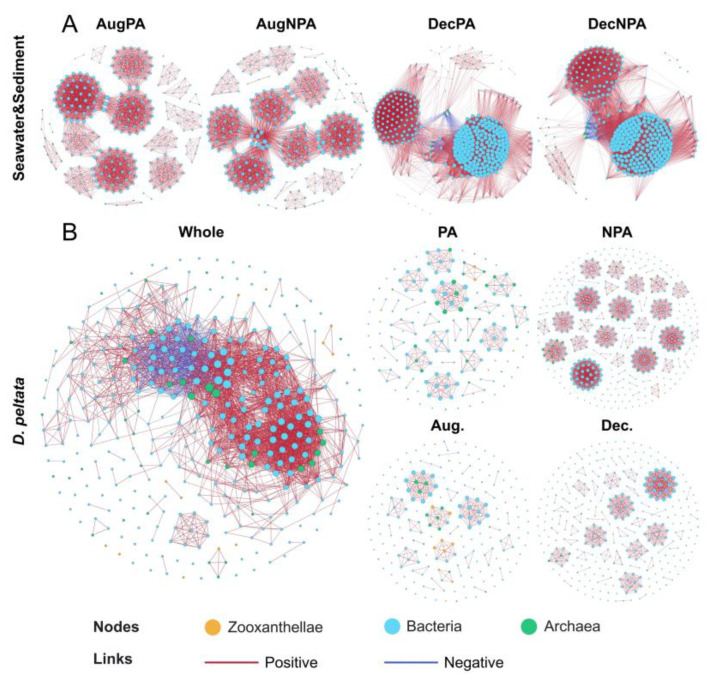
The co-occurrence networks of bacteria and archaea in the surrounding water and sediment (**A**) in the protected area and non-protected area in August (AugPA and AugNPA) and December (DecPA and DecNPA) and the co-occurrence networks of coral symbiotic zooxanthellae, bacteria, and archaea (**B**) in all coral samples (Whole), the coral samples from the protected areas (PAs) and non-protected areas (NPAs), and the coral samples in August (Aug.) and December (Dec.). Nodes are colored according to the classification, with red edges indicating theoretically positive correlations and blue edges indicating theoretically negative correlations. Connections are defined as solid correlations (Spearman’s |ρ| > 0.6) and significance (*p* < 0.01).

The network structure of the co-occurrence relationships of bacteria and archaea in the sediment and seawater in December may be more complex than that in August (Figure 7A). The network topology showed that AugNPA had more nodes and edges than DecNPA (Table S6).

A separate network analysis of *D. pelata* samples from different locations established a co-occurrence network containing zooxanthellae and bacterial and archaeal communities at the genus level (Figure 7B). As shown in Table S7, theoretically, the most relevant microorganisms were bacteria, and the relative frequencies of all dominant genera differed significantly among samples from different environments, thus potentially forming different taxa. All networks theoretically had more positive than negative connections; the positive correlation ratio was 85.21–99.87%. In addition, the modularity indices of the five co-occurrence networks were 0.448, 0.913, 0.896, 0.849, and 0.838. NPA had a higher average degree, node number, and edge number than PA in the coral microbial network. Based on the key classification principles of high average degree, high closeness centrality, and low betweenness centrality [36], Table S8 lists five potential key species or genera of *D. pelata* symbiotic microorganisms. Among these, the zooxanthellae were C36, C1228, C3n, C1a, and C1c; the main bacterial phylum was Bacteroidetes, and the main archaeal phylum was Nanoarchaeota.

## 4. Discussion

### 4.1. Coral Symbiotic Microorganisms

Symbiodiniaceae has been partitioned into multiple genera to better reflect their origins and divergence, as determined by molecular dating [37]. Many studies have shown that corals are associated primarily with endosymbionts in the genus *Cladocopium* (Clade C) [38,39]. This is mainly because of the robust adaptability of *Cladocopium* to its surroundings, particularly the thermally resilient lineage known as *Cladocopium* C15, which is commonly observed in *Porites* species [40]. The data collected in this study clearly show that in the Symbiodiniaceae family of *D. peltata*, *Cladocopium* dominated in different environments, with C1 being particularly prominent [29]. C1 is widely distributed in the waters of northern China, Okinawa, and the Jeju islands. These areas are typically characterized by low sea temperatures and high human activity levels [41,42,43]. Therefore, *D. peltata* may have established a stable symbiotic relationship with C1 to adapt to subtropical environments. Studies have also found that the symbiosis between host corals and Symbiodiniaceae changes over time and space on a small scale, with environmental differences [44,45]. Although seasonal changes have little effect on the composition of symbiotic-dominant algae [46], the diversity of non-dominant algae living in symbiosis with corals is affected by seasonal changes [47]. In the present study, C1, C1c, C18, C3, and C1054 were consistently present in different environments, indicating that they were the dominant algae in *D. peltata*. However, the diversity of Symbiodiniaceae in December was lower than in August, suggesting that corals suffered from stress in December and lost some Symbiodiniaceae algae. One notable difference was that C1a was found only in AugNPA, which may indicate that higher sea surface temperatures positively affect coral Symbiodiniaceae diversity [48]. However, the specific mechanism underlying the change in the coral Symbiodiniaceae requires further experimental data.

Despite the high level of bacterial diversity in corals, the dominant bacteria (relative abundance > 1%) show some stability in space and time [18]. We found that Proteobacteria, Bacteroidetes, and Cyanobacteria in *D. peltata* were the dominant bacterial species, consistent with the overall composition of coral bacteria worldwide [49,50]. As the surrounding environment changes, the symbiotic bacteria composition within corals undergoes dynamic changes [25]. For example, SAR324_ clade Marine _group_B, widely present in deep seawater (75–125 m), has highly plastic metabolic characteristics, including fixing inorganic carbon and metabolizing oxidized sulfur [51]. In the present study, this bacterium only appeared in AugPA, indicating that *D. peltata* may have carried out more active carbon metabolism and sulfide oxidation in this area. Environmental factors can significantly influence microbial composition and function within a specific location [52]. The NMDS diversity analysis results revealed significant differences in the ecological structures of the PA and the NPA. The Mantel test analysis revealed that nitrogen nutrients are essential in distinguishing the PA from the NPA. The alpha diversity results showed that bacteria in the NPA had a higher Shannon index than those in the PA (*p* < 0.01), which is consistent with the increase in the microbial richness of coral reefs when facing stress [53], possibly because opportunistic bacteria take advantage of compromised immune systems to colonize corals [54]. Studies have shown that some bacteria enhance stress resistance in coral hosts [55]. For example, Firmicutes are a typical class of beneficial bacteria essential in improving corals’ stress tolerance [56]. This study revealed that in August or December, the abundance of Firmicutes in *D. peltata* in the NPA was higher than in the PA. This may be a beneficial bacterial adjustment by the coral in the face of environmental stress. This study identified *Nisaea* in AugPA as a biomarker [57], and *Thalassotalea*, *Vibrio*, and *Woeseia* pathogenic bacteria were found in AugNPA, DecPA, and DecNPA [58,59,60]. However, the beneficial bacteria *Spiroplasma*, *Endozoicomonas*, and *Bacillus* are still used as biomarkers in these three areas [55]. These results show that the AugNPA, DecPA, and DecNPA environments have a specific stress effect on corals. However, corals actively adjust their bacterial community structure while adapting to the environment, with beneficial bacteria being the dominant species. This indicates the independent adaptability of corals to their environment.

Coral-associated archaea are often overlooked in genomic studies of coral microorganisms, primarily because of their low read count in genome sequencing compared to bacteria [61]. Furthermore, primers commonly used to target bacterial 16S rRNA genes often fail to detect archaeal 16S rRNA sequences [62]. Nonetheless, studies have shown that Archaea play a vital role in the nitrogen cycle in coral symbiotic functional systems [63], mainly through nitrification and denitrification processes, which help the mucus layer capture excess ammonium and thus become a nutrient sink [21]. Therefore, studying the coral archaea is crucial. This study’s analysis of the coral archaea showed that the phylum Crenarchaeota dominated the coral archaea. According to previous studies, the average proportion of Crenarchaeota in the environment is 79% [21], which is consistent with the results of this study. NMDS and mantle test analyses of archaea revealed that nitrogen content is an essential indicator of the composition of archaeal communities. Temperature was not a critical factor affecting microbial composition, possibly due to the adaptation of *D. peltata* to a lower temperature range. Although our understanding of archaeal function remains limited, genomic analyses have demonstrated their potential for nitrogen cycling, particularly the ammonium oxidation capabilities of Crenarchaeota [40]. Halobacteria typically exist in high-salt environments and adapt well to high salt concentrations [64]. A certain number of halobacteria were detected in the sampling area in August, consistent with the environmental parameters. In the December sampling area, methanogens such as *Methanobrevibacter*, *Methanobacterium*, and *Methanococcoides* were dominant. This indicates that the sampling area in December may be rich in organic waste or decomposition products of organic matter and may be anaerobic [65]. Coral symbiotic microorganisms participate in the circulation of nutrients and the maintenance of productivity, which is beneficial to the stability of the entire coral reef ecosystem [56].

### 4.2. Effects of Environmental Factors on Symbiotic Microorganisms

The core coral microbiome is crucial in adapting coral holobionts to environmental stresses [66]. The abundance of symbiotic coral microorganisms often changes in response to environmental influences [55]. In this study, the core zooxanthellae C1 ranged from 56.25% in AugPA to 44.22% in AugNPA, indicating that the relative abundance of core Symbiodiniaceae changed with environmental changes (Figure 2B). In a study of coral symbiotic microorganisms at different latitudes in China, the core group of symbiotic algae in the same coral changed at different latitudes [29]. The core bacteria SAR324_cladeMarine_group_B of the corals in this study ranged from 41.98% of AugPA to 0.23% of AugNPA, indicating that the coral core bacterial community changed from dominant bacterial species to rare bacterial species (Figure 4A). In this study, the core archaea Crenarchaeota of corals ranged from 90.67% in AugPA to 95.58% in AugNPA, indicating that the relative abundance of core archaea also changed with environmental changes (Figure 5C). However, its change sensitivity was lower than that of bacteria. Higher DIN may have caused the shift in the centrality of coral symbiont microorganisms in this study in AugNPA. Furthermore, in the face of environmental changes, core symbiotic microorganisms may interact synergistically to provide nutritional support for coral holobionts to adapt to their environments [67].

Environmental factors such as high nutrient salinity may drive core coral microbial communities from dominant to rare [68]. Climate change could disrupt the symbiotic relationship between corals and their symbiotic microorganisms, causing major changes in the composition of coral microbial communities, characterized by overgrowth of pathogenic microorganisms, which will lead to coral bleaching and/or disease [69]. In summary, the impact of environmental factors on coral symbiotic microbial communities is a relatively complex process involving the relative abundance of core microbial community members, the importance of rare microbial species, and the role of environmental factors. A better understanding of these effects will benefit the conservation and management of coral ecosystems.

### 4.3. Co-Occurrence Network Features

Co-occurrence network analysis is widely used to analyze the interactive relationships of microbial communities. It provides an overall insight into the composition of microbial communities and a better understanding of the complex interactions between microorganisms [70,71]. The seawater and sediment network diagrams and topological structures (Figure 7A and Table S6) showed that AugNPA had a higher network complexity than AugPA, consistent with the network results for coral symbiotic microorganisms. Meanwhile, the higher number of nodes and a lower average degree in NPA indicated that the node connections in the network were relatively dispersed rather than concentrated on a few nodes. This shows lower network stability and less efficient information dissemination [72], indicating the vulnerability of seawater and sediment ecosystems in NPA. DecPA also had a higher network density and lower average degree, suggesting that DecPA has a similar ecological vulnerability to AugNPA. The differences in the microbial network relationships between PA and NPA illustrate the importance of establishing PA to maintain the balance and strength of marine ecosystems. To utilize the interactions between microorganisms to improve the stability of marine ecosystems, it is necessary to explore the experimental conditions by creating a suitable environment.

We constructed a symbiotic community network based on zooxanthellae, bacteria, and archaea among coral symbiotic microorganisms. The results showed that the co-occurrence relationships of bacteria in different environments were complex and diverse, occupying a significant position in the co-occurrence network. In contrast, coral symbiotic zooxanthellae and archaea co-occurrence patterns were relatively stable. The topology of a network can visually represent the interactions between microorganisms [73]. In this study, the NPA had more nodes and edges and a higher network density and degree than the PA. The microbial network was more connected than the microbial network in December than in August. This suggests that coral microbial communities with more significant anthropogenic impact have more complex co-occurrence networks. Modularity represents the degree of microbial aggregation in blocks and is an essential indicator of microbial interactions [74]. All classified network structures had high modularity values (0.448–0.913), all greater than 0.4, indicating an apparent modular structure [75]. This modular feature helps reduce the impact of environmental chaos, with each module containing one or more internally tightly connected microbial groups [76]. The whole and December groups exhibited high-density but low-modular network structures that may be unstable under external interference [77]. Corals from the PA were more modular than those from the NPA, indicating that the PA microorganisms may gather more closely and be able to adjust more efficiently to changes in the external environment [78]. These results suggest that coral microbial networks heavily disturbed by humans are more fragile and unstable than those less disturbed [79]. In this study, most microbial interactions were positive across all networks. Positive microbial interactions can form cooperative or symbiotic alliances, allowing symbiotic coral microorganisms to exchange nutrients rapidly. However, intense positive interactions are often accompanied by significant internal environmental pressures, resulting in fragile internal coral environments of corals [80]. Therefore, positive interactions within coral microbial communities may harm coral hosts in areas of severe anthropogenic disturbance, even though they increase network stability. Further studies should be conducted to elucidate the mechanisms by which nutrient enrichment enhances interactions between coral symbiotic microorganisms.

In summary, PA corals have a relatively stable microbial community network that helps to maintain homeostasis in their internal environment. Under environmental pressures, such as high temperature, high acidity, and eutrophication, the immunity of corals decreases, and the composition of the symbiotic microbial community undergoes significant changes. Some pathogenic bacteria invade the internal symbiotic environment of corals and cause coral bleaching [81]. Coral microbial diversity increases under environmental stress, enhancing microbial interactions and establishing relationships between adjacent organisms, leading to increased correlation, reduced stability, and reduced host adaptability [29]. This study provides an in-depth exploration of the possible impact of human factors on the symbiotic microbial interactions of *D. peltata* by creating a co-occurrence network. These results indicate that interactions between coral microbial communities become more complex under severe anthropogenic disturbance.

### 4.4. Composition of Coral Keystone Taxa

Microbial interactions are key factors that determine the community structure and function [82]. In microbial communities, some key taxa may have low abundance in space and time but have a significant impact on the structure and function of the microbiome, either individually or as a group. These taxa play unique and critical roles in microbial communities and, if removed, lead to significant changes in microbial community structure and function [36]. This study identified some possible key groups of *D. peltata* symbiotic microorganisms using co-occurrence network analysis (Table S8). These results indicated that not all keystone zooxanthellae taxa were dominant. However, these keystone taxa mainly exist in the NPA, and it can be inferred that the NPA has more complex food webs, symbiotic relationships, and ecosystem services [36]. Keystone taxa, among bacteria and archaea, play essential roles in the decomposition and transformation of organic matter, emphasizing their positive impact on material circulation and energy flow throughout the system.

## 5. Conclusions

This study analyzed the differences in the microbial community composition, influencing factors, and co-occurrence patterns in *D. peltata*, seawater, and sediments under different anthropogenic disturbances. Anthropogenic disturbances reduced the diversity of coral symbiotic algae, increased the species composition of coral symbiotic bacteria, and altered the community structure of coral archaea. DIN is a key factor affecting coral microbial communities. Co-occurrence network analysis revealed the interactions and functions of the coral microbial communities, reflecting the interactions and connections between microbial taxa in large seawater, coral, and sediment environments. These findings suggest that coral microbiomes vary across environments, which may impair host immunity, enabling diverse opportunistic bacteria in coral symbionts. Also, coral keystone taxa do not necessarily coincide with their dominant taxa. This study expands our understanding of the effect of nutrient enrichment on *D. peltata* symbiotic microorganisms (zooxanthellae, bacteria, and archaea). It improves our understanding of the adaptive mechanisms of coral holobionts in response to environmental changes. Further research is needed to fully understand the structure and function of microbial communities in coral holobionts and to determine how microorganisms in the natural environment establish connections and interact with corals.

## Figures and Tables

**Table 1:** Physico-chemical parameters of the surrounding seawater and sediment at coral sampling sites from the protected and non-protected areas in August and December.

	August Protected Area	August Non-Protected Area	December Protected Area	December Non-Protected Area
TOC (g/kg) ^1^	2.83	5.11	4.37	14.52
TP (g/kg) ^1^	0.39	0.48	0.36	1.09
TN (g/kg) ^1^	0.66	0.86	0.47	1.38
Turbidity (NTU) ^2^	3.55 ± 1.35	6.10 ± 2.25	4.84 ± 2.26	9.63 ± 2.94
DO (mg/L) ^2^	7.17 ± 0.72	4.36 ± 0.60	7.28 ± 0.37	6.47 ± 0.35
Chl a (μg/L) ^2^	5.26 ± 2.26	2.04 ± 1.10	4.12 ± 2.75	1.73 ± 0.40
Salinity (‰) ^2^	33.58 ± 0.39	33.27 ± 0.86	32.36 ± 0.15	32.46 ± 0.08
pH ^2^	8.10 ± 0.04	7.89 ± 0.05	8.20 ± 0.03	8.08 ± 0.03
Temperature (°C) ^2^	25.92 ± 0.69	27.08 ± 0.72	21.97 ± 0.52	21.79 ± 0.96
DIN (μM) ^3^	6.38	11.21	23.51	23.02
PO_4_^3−^-P (μM) ^3^	0.54	0.74	1.35	1.39
N/P ^3^	11.81	15.15	17.41	16.56
NO_3_^−^-N (μM) ^3^	3.55	5.66	22.64	21.46
NO_2_^−^-N (μM) ^3^	0.55	0.51	0.26	0.42
NH_4_^+^-N (μM) ^3^	2.28	5.04	0.61	1.14

^1^ Sediment nutrient levels were measured in the lab. ^2^ The adjacent buoys monitored seawater’s physical and chemical parameters within a month. ^3^ Seawater nutrient levels were measured in the lab.

## Data Availability

The original contributions presented in the study are included in the article/Supplementary Materials, further inquiries can be directed to the corresponding authors.

## Supplementary Materials

The following supporting information can be downloaded at: https://www.mdpi.com/article/10.3390/microorganisms12081540/s1, Table S1: The amplification primers for microorganisms in coral, sediment, and seawater; Table S2: Potential eutrophication assessment model; Table S3: The alpha diversity indices of *Duncanopsammia peltata* zooxanthellae communities in the August protected area (AugPA), August non-protected area (AugNPA), December protected area (DecPA) and December non-protected area (DecNPA); Table S4: The alpha diversity indices of *Duncanopsammia peltata* bacterial communities in the August protected area (AugPA), August non-protected area (AugNPA), December protected area (DecPA) and December non-protected area (DecNPA); Table S5: The alpha diversity indices of *Duncanopsammia peltata* archaeal communities in the August protected area (AugPA), August non-protected area (AugNPA), December protected area (DecPA) and December non-protected area (DecNPA); Table S6: Topological characteristics of microbial community co-occurrence networks at the genus level in seawater and sediment in the August protected areas (AugPAs), August non-protected areas (AugNPAs), December protected areas (DecPAs), and December non-protected areas (DecNPAs); Table S7: Topological characteristics of the co-occurrence network of microbial communities at the coral genus level in all samples (Whole), protected areas (PAs), non-protected areas (NPAs), August (Aug.), and December (Dec.); Table S8: Potential keystone genera in different sites of *Duncanopsammia* peltate; Figure S1: Shannon index of bacteria and archaea in sediments and seawater. Sediment (A); seawater (B). August protected area (AugPA), August non-protected area (AugNPA), December protected area (DecPA), and December non-protected area (DecNPA); Figure S2: Sediment species composition of bacteria and archaea in AugPA, AugNPA, DecPA, and DecPA. Bacteria (A); archaea (B). August protected area (AugPA), August non-protected area (AugNPA), December protected area (DecPA), and December non-protected area (DecNPA); Figure S3: Seawater species composition (A), linear discriminant analysis (B), and differential analysis (C) of bacteria in AugPA, AugNPA, DecPA, and DecNPA. August protected area (AugPA), August non-protected area (AugNPA), December protected area (DecPA), and December non-protected area (DecNPA); Figure S4: Seawater species composition (A), linear discriminant analysis (B), and differential analysis (C) of archaea in AugPA, AugNPA, DecPA, and DecNPA. August protected area (AugPA), August non-protected area (AugNPA), December protected area (DecPA), and December non-protected area (DecNPA); Figure S5: Non-metric multidimensional scale (NMDS) plots and PCoA analysis based on Bray–Curtis distance of sediment bacteria and archaea corresponding to ASV levels in AugPA, AugNPA, DecPA, and DecNPA. Bacterial NMDS analysis (A); archaea NMDS analysis (B); bacterial PCoA analysis (C); archaea PCoA analysis (D); August protected area (AugPA), August non-protected area (AugNPA), December protected area (DecPA), and December non-protected area (DecNPA); Figure S6: Non-metric multidimensional scale plots of seawater bacteria and archaea corresponding to ASV levels in DecPA and DecNPA. Bacteria (A); archaea (B). December protected area (DecPA), and December non-protected area (DecNPA); Figure S7: Coral symbiotic archaea Shannon index. August (A); December (B). August protected area (AugPA) and non-protected area (AugNPA); December protected area (DecPA) and non-protected area (DecNPA). References [35,83,84,85,86,87,88,89] are cited in the Supplementary Materials.
