# Supplementary material for: Impact of Nutrient Enrichment on Community Structure and Co-Occurrence Networks of Coral Symbiotic Microbiota in Duncanopsammia peltata: Zooxanthellae, Bacteria, and Archaea
Source: Microorganisms. 2024 Jul 27;12(8):1540. doi: 10.3390/microorganisms12081540 (PMC11356306; doi:10.3390/microorganisms12081540)
Supplement: Supplementary file 1 [file microorganisms-12-01540-s001.zip › microorganisms-3082239-supplementary.pdf]

Supplementary Material

Impact of nutrient enrichment on community structure and co-occurrence networks of coral symbiotic microbiota in *Duncanopsammia peltata*: Zooxanthellae, bacteria, and archaea

Chuanzhu Bai [1,2], Qifang Wang [2], Jinyan Xu [3], Han Zhang [2], Yuxin Huang [1,2], Ling Cai [2,4*], Xinqing Zheng [2,4,5] and Ming Yang [1,*]

1 *School of Environmental and Chemical Engineering, Shanghai University, Shanghai 200444, China; 15779226849@shu.edu.cn (C.B.); huangyuxin0120@163.com (Y.H.)*

2 *Key Laboratory of Marine Ecology Conservation and Restoration, Third Institute of Oceanography,*

Ministry of Natural Resources, Xiamen 361005, China; wangqifang@tio.org.cn (Q.W.);

202120949@mail.sdu.edu.cn (H.Z.); zhengxinqing@tio.org.cn (X.Z.)

3 *Fujian Key Laboratory of Island Monitoring and Ecological Development (Island Research Center, MNR), Pingtan 350400, China; xujy_liesmars@whu.edu.cn*

4 *Observation and Research Station of Island and Coastal Ecosystems in the Western Taiwan Strait, Ministry of Natural Resources, Xiamen 361005, China*

5 *Fujian Provincial Station for Field Observation and Research of Island and Coastal Zone, Zhangzhou 363216, China*

* *Correspondence: cailing@tio.org.cn (L.C.); mingyang@shu.edu.cn (M.Y.)*

This file contains 8 tables and 7 figures.

Table S1 The amplification primers for microorganisms in coral, sediment, and seawater

Subjects	Microorganism	Primers	Primer Sequence (5' - 3')	Reference
Coral	Zooxanthellae	ITS-DINO	GTGAATTGCAGAACTCCGTG	[1]
		ITS2Rev2	CCTCCGCTTACTTATATGCTT	
	August bacteria	27F	AGRGTTYGATYMTGGCTCAG	[2,3]
		1492R	RGYTACCTTGTTACGACTT	
	December bacteria	341F	CCTACGGGNGGCWGCAG	[4]
		806R	GGACTACHVGGGTATCTAAT	
	August archaea	524F	TGYCAGCCGCCGCGGTAA	[5]
		958R	YCCGGCGTTGAVTCCAATT	
	December archaea	515F	GTGCCAGCMGCCGCGGTAA	[6]
		907R	CCGTCAATTCCTTTGAGTTT	
Seawater & Sediment	Bacteria	338F	ACTCCTACGGGAGGCAGCA	[7]
		806R	GGACTACHVGGGTWTCTAAT	
	Archaea	524F	TGYCAGCCGCCGCGGTAA	[5]
		958R	YCCGGCGTTGAVTCCAATT	

Table S2 Potential eutrophication assessment model [8]

Level	Eutrophication degree	DIN (μM)	DIP (μM)	N/P
1	Poor nutrition	<14.29	<0.48	/
2	Medium nutrition and P limiting	14.29-21.43	/	⩾16
	Medium nutrition and N limiting	/	0.48-0.97	<16
3	Medium nutrition	14.29-21.43	0.48-0.97	8-30
4	Potential eutrophication and P limiting	>21.43	/	⩾16
	Potential eutrophication and N limiting	/	>0.97	<16
5	Light eutrophication	21.43-28.57	0.97-1.45	8-30
6	Medium eutrophication and P limiting	>28.57	/	⩾16
	Medium eutrophication and N limiting	/	>1.45	<16
7	Medium eutrophication	28.57-35.71	1.45-1.94	8-30
8	Heavy eutrophication and P limiting	>35.71	/	⩾16
	Heavy eutrophication and N limiting	/	>1.94	<16
9	Heavy eutrophication	>35.71	>1.94	/

Table S3 The alpha diversity indices of *Duncanopsammia peltata* zooxanthellae communities in August protected area (AugPA), August non-protected area (AugNPA), December protected area (DecPA) and December non-protected area (DecNPA)

Alpha diversity	AugPA *D. peltata*				AugNPA *D. peltata*				DecPA *D. peltata*				DecNPA *D. peltata*			
	1st	2nd	3rd	Mean ± SD	1st	2nd	3rd	Mean ± SD	1st	2nd	3rd	Mean ± SD	1st	2nd	3rd	Mean ± SD
No. of reads	13204	17516	24796	18505±5859	10978	14499	9399	11625±2611	10455	11769	15633	12619±2692	10119	11863	12624	11535±1284
ASVs	23	25	24	24±1	22	22	19	21±2	15	19	18	17±2	13	18	20	17±4
Chao1	23	25	24	24±1	22	22	19	21±2	15	19	18	17±2	13	18	20	17±4
Shannon	1.92	2.17	2.10	2.06±0.129	1.80	2.13	1.94	1.96±0.166	2.00	1.96	2.00	1.99±0.0230	1.69	1.94	1.93	1.85±0.142

Table S4 The alpha diversity indices of *Duncanopsammia peltata* bacterial communities in August protected area (AugPA), August non-protected area (AugNPA), December protected area (DecPA) and December non-protected area (DecNPA)

Alpha diversity	AugPA *D. peltata*				AugNPA *D. peltata*				DecPA *D. peltata*				DecNPA *D. peltata*			
	1st	2nd	3rd	Mean ± SD	1st	2nd	3rd	Mean ± SD	1st	2nd	3rd	Mean ± SD	1st	2nd	3rd	Mean ± SD
No. of reads	44100	29914	35821	36612±7126	43463	44178	28729	38790±8720	14326	22664	20888	19293±4392	48716	50057	62981	53918±7877
ASVs	278	229	354	287±63	1457	545	925	976±458	518	530	571	540±28	3504	3047	2745	3099±382
Chao1	277	229	351	286±61	1471	550	925	982±463	518	558	576	551±30	3588	2980	2536	3035±528
Shannon	2.64	2.56	1.87	2.36±0.423	5.08	3.88	4.56	4.51±0.602	4.65	4.41	4.62	4.56±0.131	6.93	6.98	7.14	7.02±0.110

Table S5 The alpha diversity indices of *Duncanopsammia peltata* archaeal communities in August protected area (AugPA), August non-protected area (AugNPA), December protected area (DecPA) and December non-protected area (DecNPA)

Alpha diversity	AugPA *D. peltata*				AugNPA *D. peltata*				DecPA *D. peltata*				DecNPA *D. peltata*			
	1st	2nd	3rd	Mean ± SD	1st	2nd	3rd	Mean ± SD	1st	2nd	3rd	Mean ± SD	1st	2nd	3rd	Mean ± SD
No. of reads	12017	8352	6520	8963±2799	9797	10299	11215	10437±719	66967	65657	68277	66967±1310	46865	51962	40363	46397±5814
ASVs	41	22	36	33±10	35	40	88	54±29	270.5	338	203	271±68	436	700	462	533±145
Chao1	41	22	36	33±10	35	40	88	54±29	273	340	207	273±67	434	707	462	534±150
Shannon	2.36	2.53	2.96	2.62±0.309	2.34	3.17	3.01	2.84±0.440	3.24	3.47	3.01	3.24±0.230	4.83	4.39	4.86	4.69±0.263

Table S6 Topological characteristics of microbial community co-occurrence networks at the genus level in seawater and sediment in August protected areas (AugPA), August non-protected areas (AugNPA), December protected areas (DecPA), and December non-protected areas (DecNPA)

	AugPA	AugNPA	DecPA	DecNPA
Total nodes	264	270	367	328
Proportion bacteria and archaea nodes	240:24	241:29	338:29	294:34
Total edges	3372	3441	22281	21496
Average degree	25.545	25.489	121.422	131.073
Graph density	0.097	0.095	0.332	0.401
Modularity	0.726	0.717	0.358	0.292
Positive links	100.00%	100.00%	98.43%	98.49%

Table S7 Topological characteristics of the co-occurrence network of microbial communities at the coral genus level in all samples (Whole), protected areas (PA), non-protected areas (NPA), August (Aug.), and December (Dec.)

	Whole	PA	NPA	Aug.	Dec.
Total nodes	349	169	465	214	356
Proportion zooxanthellae, bacteria and archaea nodes	12:298:39	10:128:31	10:419:36	14:179:21	8:319:29
Total edges	3003	304	2324	247	748
Average degree	17.209	3.598	9.996	2.308	4.202
Graph density	0.049	0.021	0.022	0.011	0.012
Modularity	0.448	0.913	0.896	0.849	0.838
Positive links	85.21%	99.67%	99.87%	99.60%	98.40%

Table S8 Potential keystone genera in different sites of *Duncanopsammia peltata*

Zooxanthellae (Species&Degree)	Bacteria (Genus&Phylum&Degree)	Archaea (Genus&Phylum&Degree)
C36,3	unclassified_f__Rhodothermaceae,Bacteroidota,71	unclassified_o__Woesearchaeales,Nanoarchaeota,71
C1229,2	unclassified_f__Bacteroidetes_BD2-2,Bacteroidota,71	unclassified_f__SCGC_AAA011-D5,Nanoarchaeota,70
C3n,2	unclassified_c__Thermodesulfovibrionia,Nitrospirota,66	unclassified_c__Bathyarchaeia,Crenarchaeota,56
C1a,2	unclassified_o__Gammaproteobacteria_Incertae_Sedis,Proteobacteria,63	unclassified_c__Odinarchaeia,Asgardarchaeot,48
C1c,1	unclassified_f__Desulfobulbaceae,Desulfobacterota,62	unclassified_c__Thermoplasmata,Thermoplasmatota,48

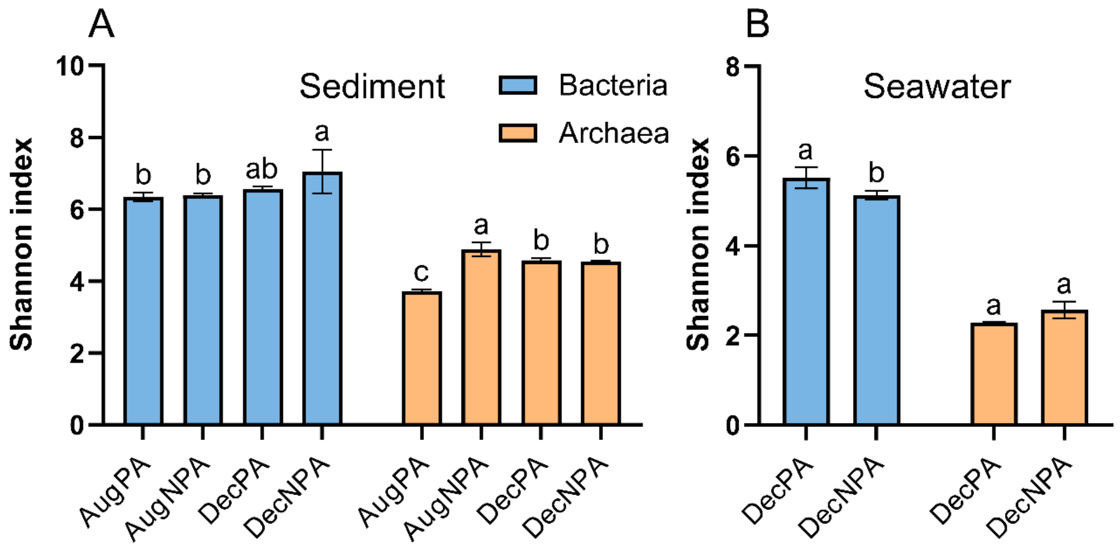

Figure S1. Shannon index of bacteria and archaea in sediments and seawater. Sediment (A); Seawater (B). August protected area (AugPA), August non-protected area (AugNPA), December protected area (DecPA) and December non-protected area (DecNPA).

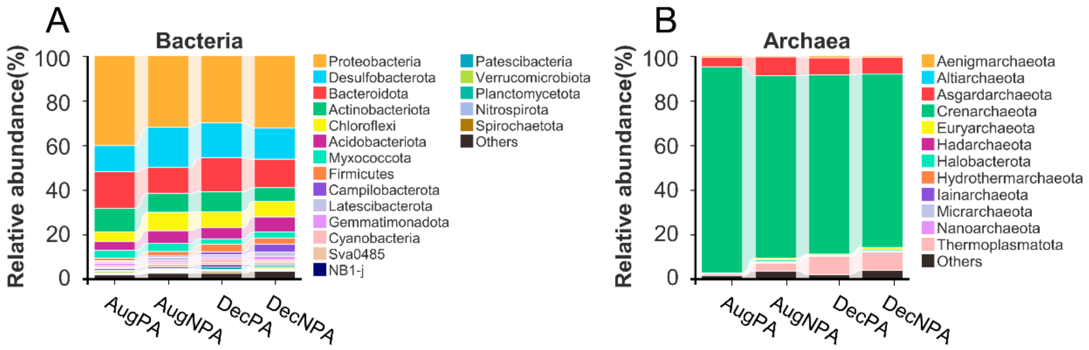

Figure S2. Sediment species composition of bacteria and archaea in AugPA, AugNPA, DecPA, and DecPA. Bacteria (A); Archaea (B). August protected area (AugPA), August non-protected area (AugNPA), December protected area (DecPA) and December non-protected area (DecNPA).

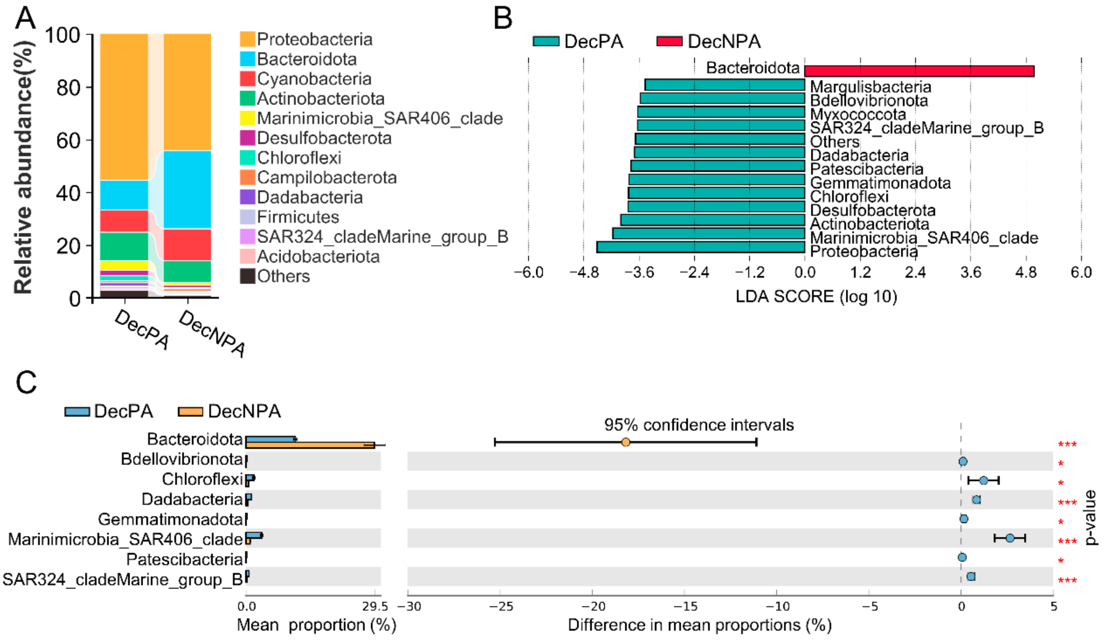

Figure S3. Seawater species composition (A), linear discriminant analysis (B), and differential analysis (C) (***: $p < 0.001$, *: $p < 0.05$) of bacteria in AugPA, AugNPA, DecPA,and DecNPA. August protected area (AugPA), August non-protected area (AugNPA), December protected area (DecPA) and December non-protected area (DecNPA).

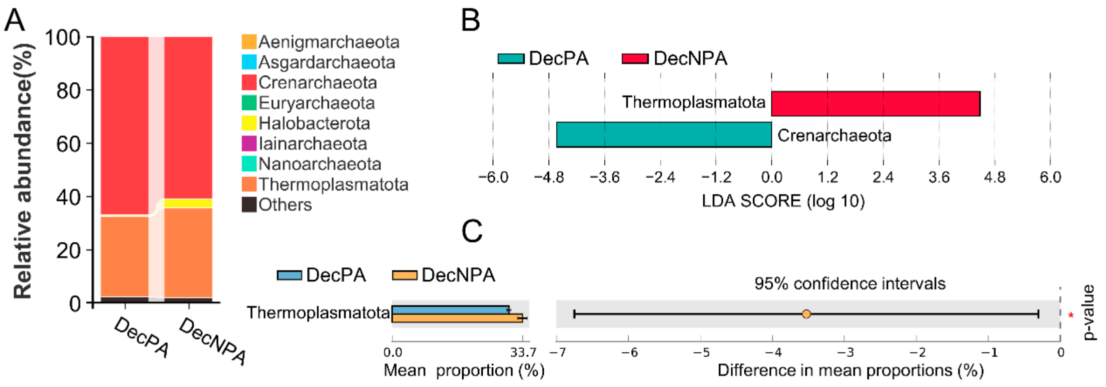

Figure S4. Seawater species composition (A), linear discriminant analysis (B), and differential analysis (C) (*: $p < 0.05$)of archaea in AugPA, AugNPA, DecPA,and DecNPA. August protected area (AugPA), August non-protected area (AugNPA), December protected area (DecPA) and December non-protected area (DecNPA).

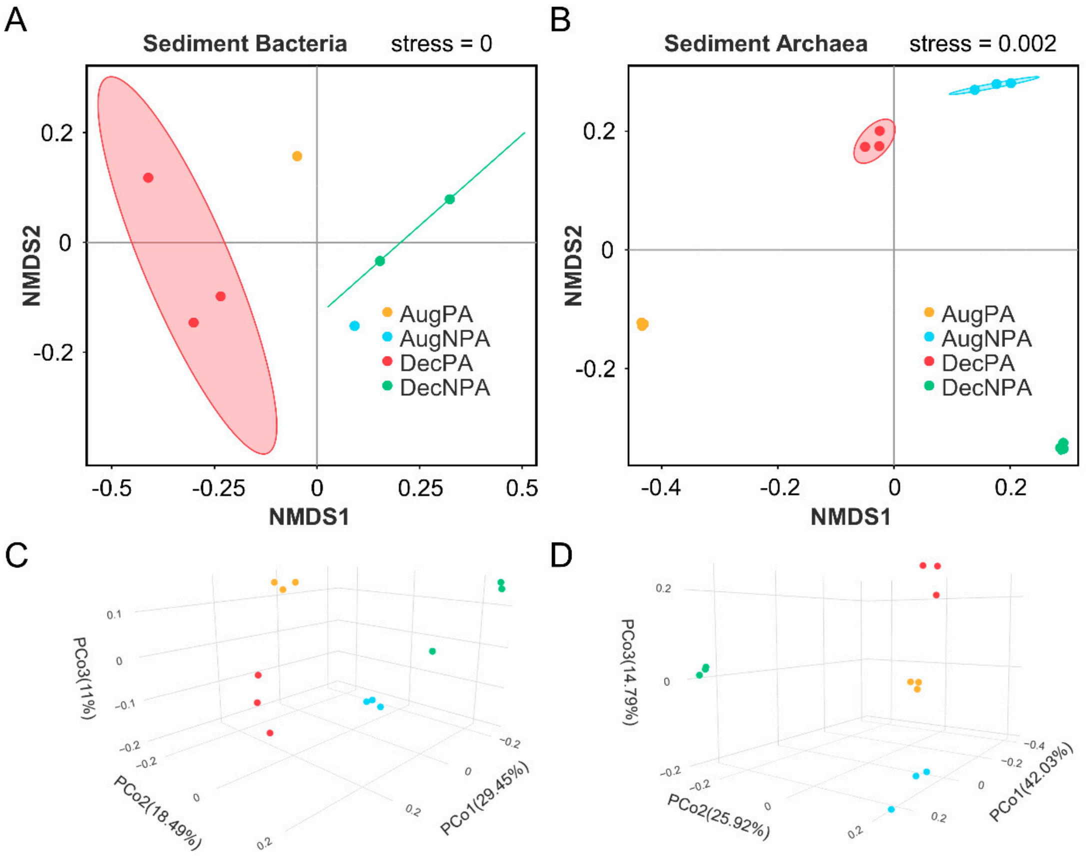

Figure S5. Non-metric multidimensional scale (NMDS) plots and PCoA analysis based on Bray-Curtis distance of sediment bacteria and archaea corresponding to ASV levels in AugPA, AugNPA, DecPA, and DecNPA. Bacterial NMDS analysis (A); Archaea NMDS analysis (B); Bacterial PCoA analysis (C); Archaea PCoA analysis (D); August protected area (AugPA), August non-protected area (AugNPA), December protected area (DecPA) and December non-protected area (DecNPA).

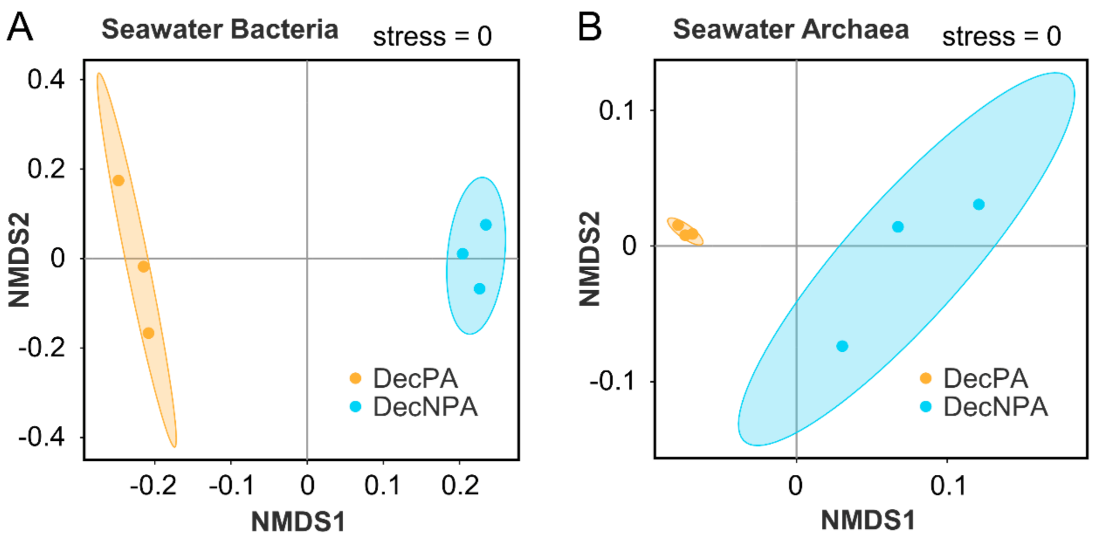

Figure S6. Non-metric multidimensional Scale plots of seawater bacteria and archaea corresponding to ASV levels in DecPA and DecNPA. Bacteria (A); Archaea (B).December protected area (DecPA) and December non-protected area (DecNPA).

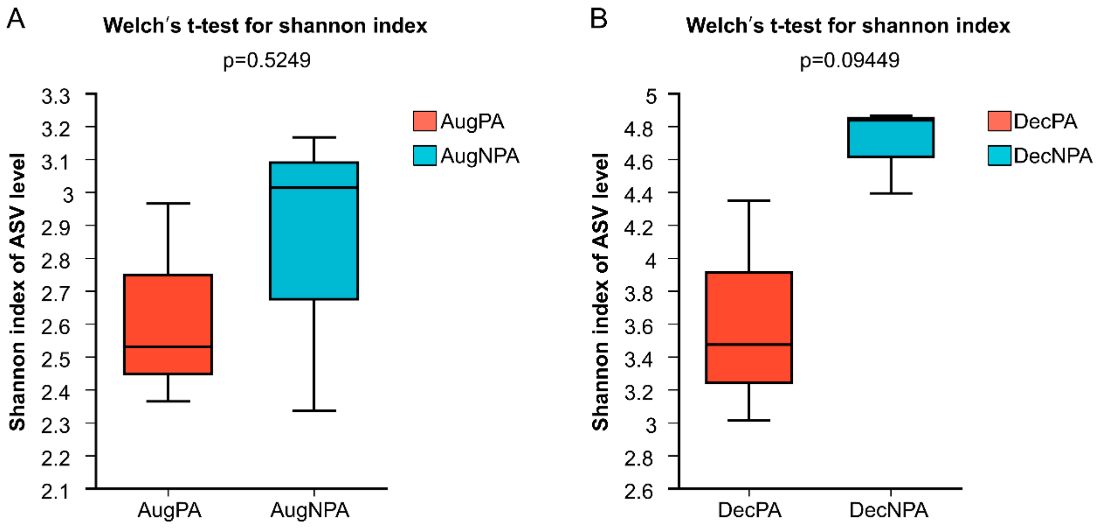

Figure S7. Coral symbiotic archaea Shannon index. August (A); December (B). August protected area (AugPA) and non-protected area (AugNPA); December protected area (DecPA) and non-protected area (DecNPA).

References

1. Stat, M.; Pochon, X.; Cowie, R.O.M.; Gates, R.D. Specificity in Communities of Symbiodinium in Corals from Johnston Atoll. *Mar. Ecol. Prog. Ser.* **2009**, *386*, 83–96, doi:10.3354/meps08080.
2. Amann, R.I.; Ludwig, W.; Schleifer, K.H. Phylogenetic Identification and in Situ Detection of Individual Microbial Cells without Cultivation. *Microbiol. Rev.* **1995**, *59*, 143–169, doi:10.1128/mr.59.1.143-169.1995.
3. Rohwer, F.; Seguritan, V.; Azam, F.; Knowlton, N. Diversity and Distribution of Coral-Associated Bacteria. *Mar. Ecol. Prog. Ser.* **2002**, *243*, 1–10, doi:10.3354/meps243001.
4. Guo, M.; Wu, F.; Hao, G.; Qi, Q.; Li, R.; Li, N.; Wei, L.; Chai, T. Bacillus Subtilis Improves Immunity and Disease Resistance in Rabbits. *Front. Immunol.* **2017**, *8*.
5. Pires, A.C.C.; Cleary, D.F.R.; Almeida, A.; Cunha, Â.; Dealtry, S.; Mendonça-Hagler, L.C.S.; Smalla, K.; Gomes, N.C.M. Denaturing Gradient Gel Electrophoresis and Barcoded Pyrosequencing Reveal Unprecedented Archaeal Diversity in Mangrove Sediment and Rhizosphere Samples. *Appl. Environ. Microbiol.* **2012**, *78*, 5520–5528, doi:10.1128/AEM.00386-12.
6. Bobadilla Fazzini, R.A.; Levican, G.; Parada, P. Acidithiobacillus Thiooxidans Secretome Containing a Newly Described Lipoprotein Licanantase Enhances Chalcopyrite Bioleaching Rate. *Appl. Microbiol. Biotechnol.* **2011**, *89*, 771–780, doi:10.1007/s00253-010-3063-8.
7. Yu, Y.; Lee, C.; Kim, J.; Hwang, S. Group-Specific Primer and Probe Sets to Detect Methanogenic Communities Using Quantitative Real-Time Polymerase Chain Reaction. *Biotechnol. Bioeng.* **2005**, *89*, 670–679, doi:10.1002/bit.20347.
8. Chen, B.; Wang, K.; Peng, C.; Dong, X.; Wang, S.; Lin, H. Changes of Eutrophication Degree in Dongshan Bay in China Affected by the COVID-19 Outbreak. *Reg. Stud. Mar. Sci.* **2023**, *66*, 103119, doi:10.1016/j.rsma.2023.103119.
